# A Cross-Validated Feature Selection (CVFS) approach for extracting the most parsimonious feature sets and discovering potential antimicrobial resistance (AMR) biomarkers

**DOI:** 10.1016/j.csbj.2022.12.046

**Published:** 2022-12-28

**Authors:** Ming-Ren Yang, Yu-Wei Wu

**Affiliations:** a Graduate Institute of Biomedical Informatics, College of Medical Science and Technology, Taipei Medical University, Taipei 110, Taiwan, ROC; b Department of Electrical Engineering, National Taiwan University of Science and Technology, Taipei 106, Taiwan, ROC; c Clinical Big Data Research Center, Taipei Medical University Hospital, Taipei, Taiwan, ROC; d TMU Research Center for Digestive Medicine, Taipei Medical University, Taipei 110, Taiwan, ROC

**Keywords:** CVFS, cross validated feature selection, AMR, antimicrobial resistance, SVM, support vector machine, PATRIC, Pathosystems Resource Integration Center, CARD, Comprehensive Antibiotic Resistance Database, RGI, Resistance Gene Identifier, XGBoost, eXtreme Gradient Boosting, Cross-Validated Feature Selection, CVFS, Feature selection, Biomarker, Pan-genome, Antimicrobial resistance

## Abstract

Understanding genes and their underlying mechanisms is critical in deciphering how antimicrobial-resistant (AMR) bacteria withstand detrimental effects of antibiotic drugs. At the same time the genes related to AMR phenotypes may also serve as biomarkers for predicting whether a microbial strain is resistant to certain antibiotic drugs. We developed a Cross-Validated Feature Selection (CVFS) approach for robustly selecting the most parsimonious gene sets for predicting AMR activities from bacterial pan-genomes. The core idea behind the CVFS approach is interrogating features among non-overlapping sub-parts of the datasets to ensure the representativeness of the features. By randomly splitting the dataset into disjoint sub-parts, conducting feature selection within each sub-part, and intersecting the features shared by all sub-parts, the CVFS approach is able to achieve the goal of extracting the most representative features for yielding satisfactory AMR activity prediction accuracy. By testing this idea on bacterial pan-genome datasets, we showed that this approach was able to extract the most succinct feature sets that predicted AMR activities very well, indicating the potential of these genes as AMR biomarkers. The functional analysis demonstrated that the CVFS approach was able to extract both known AMR genes and novel ones, suggesting the capabilities of the algorithm in selecting relevant features and highlighting the potential of the novel genes in expanding the antimicrobial resistance gene databases.

## Highlights

• Antimicrobial resistance (AMR) can be accurately predicted from bacterial pan-genome.
• Cross-Validated Feature Selection (CVFS) approach for parsimonious feature selection.
• Potential AMR gene biomarkers can be extracted using the CVFS approach.
• Much reduced CVFS gene sets achieve comparable prediction power as other approaches.

## Graphical Abstract

**Figure:**
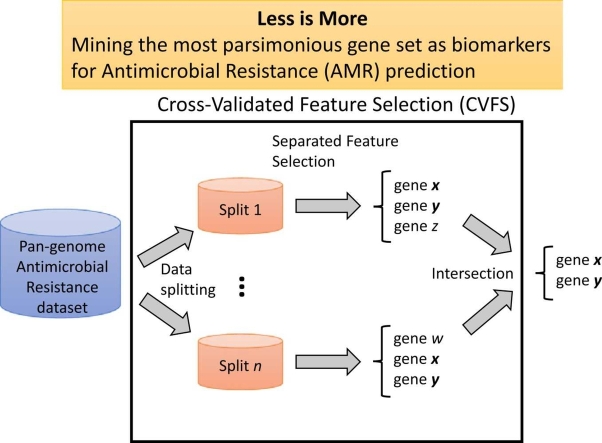

## 1 Background

Antimicrobial-resistant (AMR) pathogens are becoming one of the major threats in modern medical world. The ability of microbial pathogens to develop resistance against antibiotic drugs may render common drugs inapplicable in treating infectious diseases. Worse, the time for bacteria to develop antibiotic resistance ability is very short (as demonstrated in the famous MEGA-plate experiment [1]), outpacing the drug development speed of most pharmaceutical companies. Needless to say, it is becoming very critical to identify the antibiotic resistance profiles of the pathogens as fast and accurate as possible for hospitals to treat patients in time.

One of the promising approaches to identify or predict whether a pathogen is resistant to certain drugs is through culture-based laboratory experiments. However, the culture-based testing methods such as broth dilution or agar disk diffusion are very time-consuming [2]. An alternative method was to conduct genotyping on the pathogen, as genes were identified to play critical roles in drug resistance mechanisms. One of the earliest examples was *ampC*, in which the *ampC* gene was found to encode class C beta-lactamase for conferring beta-lactam resistance in the 1970s and 1980s [3], and the *Escherichia coli ampC* gene has been sequenced and determined in 1981 [4]. Another example was that the genotype and phenotype were highly correlated and can be used to predicting trimethoprim-sulfamethoxazole resistance in *Staphylococcus aureus* [5]. These examples demonstrated that AMR genes were correlated with and may be used for predicting resistance phenotypes. Therefore searching AMR genes for drug resistance prediction purpose and the maintenance of AMR gene databases are still very important research tasks in medicine.

Approaches for predicting whether a pathogenic strain is AMR or not based on their genetic contents have also been developed [6], [7], [8], [9], [10], [11]. Genes that are critical to the antimicrobial resistance capabilities have been identified and deposited into databases (such as CARD [12], ARDB [13], Resfam [14], or ResFinder [15]). The databases, however, may not be comprehensive since people keep digging out novel AMR genes from various environments [16], [17], [18], [19]. In addition most database-based approaches may return too many candidate AMR genes but fail to pinpoint the most crucial ones, highlighting the need to explore novel approaches for finding most crucial AMR genes from bacterial genomes.

With the growing amount of data in public databases such as the Pathosystems Resource Integration Center (PATRIC) [20], it is now possible to identify novel AMR genes by associating them with the resistance phenotypes. In our previous work we used pan-genome along with a machine learning feature selection approach to identify the most plausible gene sets for predicting AMR strains [21]; meanwhile we also showed that the gene sets that we extracted using machine learning feature selection approach achieved much higher prediction accuracies. However, the previous study only focused on the known AMR genes without considering others. Since other genes, including functionally-unknown ones, may also act as good predictors for AMR status [22], it is thus necessary to expand the search area to the entire gene set in order to identify better candidates that may act as AMR biomarkers.

In this study we developed a Cross-Validated Feature Selection (CVFS) approach for effectively and unbiasedly extracting the most parsimonious gene sets that may serve as AMR biomarkers from genomic data. As hinted by its name, the CVFS approach works by randomly splitting the entire training data into distinct sub-parts and looking for shared features that are extracted from different sub-parts. In addition this process is going to be repeated a few runs, and only features consistently identified in most of the runs are included in the final feature set. In other words, the CVFS approach has the potential to select as few features (that is, genes) as possible that may still serve as good AMR predictors. By testing the idea on pan-genomes constructed from bacterial antimicrobial resistance genomics datasets downloaded from the Pathosystems Resource Integration Center (PATRIC) database [20], we showed that our approach was able to identify dozens of genes (termed CVFS-genes hereafter, as these genes were identified using the CVFS approach) that were able to serve as very accurate predictors for the drug resistant (R) or susceptible (S) phenotypes. In one of the most extreme cases we found that 98% prediction accuracy can be achieved using only two CVFS-genes for *Salmonella enterica* amoxicillin/clavulanic acid resistance. We also conducted functional analysis to discuss the functional roles of the CVFS-genes.

## 2 Results

### 2.1 Pan-genome construction

Totally 4216 and 7712 genomes were downloaded from the PATRIC website for *Escherichia coli* and *Salmonella enterica* species, respectively. After removing low quality genomes and potential contaminated ones (119 for *E. coli* and 463 for *S. enterica*; see Methods for details), protein-coding genes from the remaining 4097 and 7249 genomes were clustered into gene clusters. Table 1 lists the statistics of the constructed pan-genomes, including the number of gene clusters and the numbers of core and accessory genes.

**Table 1:** Statistics of the bacterial pan-genome.

	*Escherichia coli*	*Salmonella enterica*
Downloaded genome number	4216	7712
Clean genome number	4097	7249
Gene cluster number	163,429	79,536
Core gene number	877	1479
Accessory gene number	162,552	78,057

**Fig. 1:**
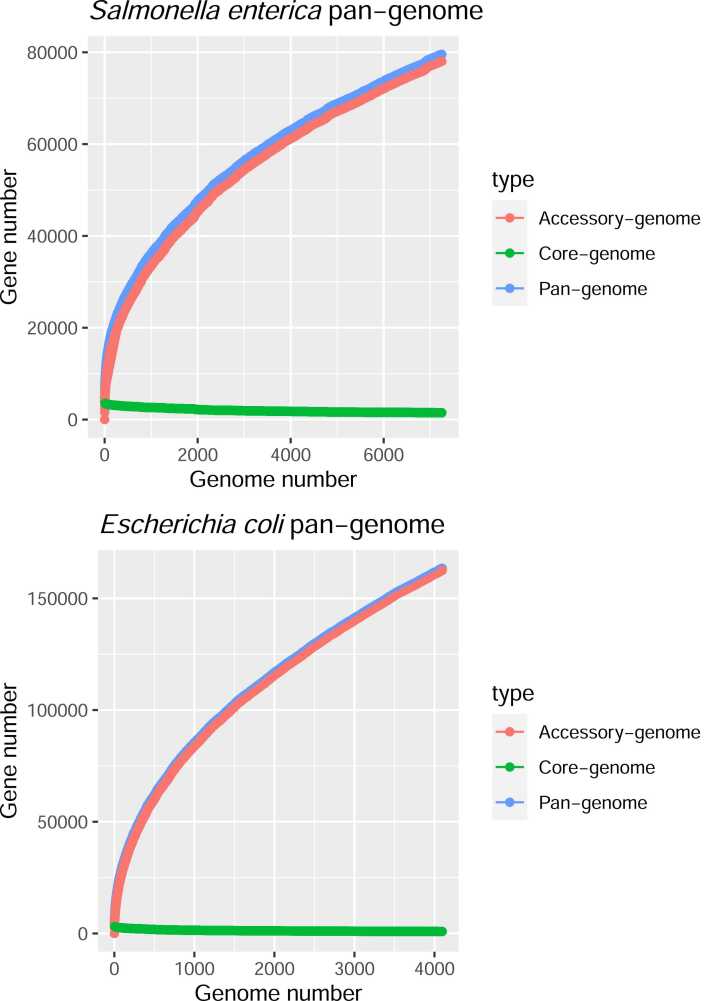
The growth curves of both *Salmonella enterica* and *Escherichia coli* core-, accessory-, and pan-genomes. X-axis indicates the number of genomes while y-axis represents the number of genes.

The pan-genome gene cluster growth curves are shown in Fig. 1, in which one can clearly see that the core genome sizes are almost stationary while the accessory genome sizes keep growing unstoppably for both species, indicating that the two pan-genomes may belong to open-pan-genome. By fitting the pan-genome growth curves to a power-law growth model, we estimated that the power law model parameters for γ are 0.47 and 0.43 for *E. coli* and *S. enterica* respectively (see Methods for details). Since a pan-genome was defined to be an open-pan-genome if γ>0, the two bacterial pan-genomes were more likely to be open-pan-genomes.

### 2.2 Cross-Validated Feature Selection (CVFS) approach

**Fig. 2:**
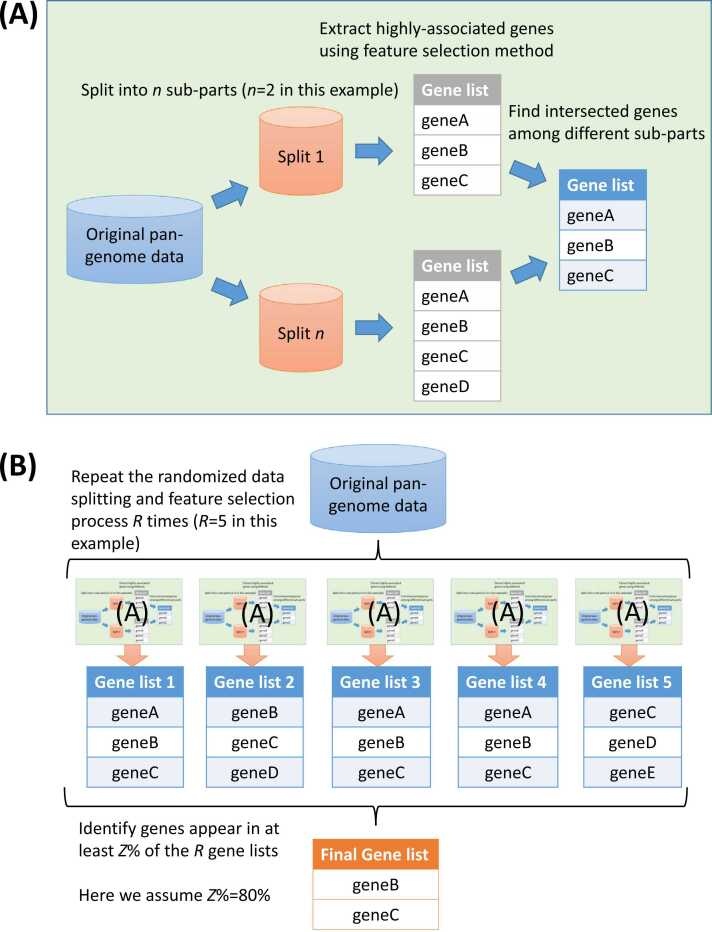
An example run of the cross-validated feature selection approach. (A) the randomized splitting of the pan-genome data into *n* subsets (*n* = 2 in this case) for feature selection and gene set intersection; (B) repeating the randomized splitting and feature selection/intersection for *R* times (*R*=5) and identify features that appear in at least *Z*% of repeated runs (*Z*%=80%).

The basic idea of cross-validated feature selection approach consists of several steps, including 1) splitting the pan-genome dataset into disjoint sub-parts, 2) conducting feature selection on each sub-part individually, 3) intersecting the features selected from all sub-parts, and 4) repeating steps 1–3 several times to identify features consistently appear in most of the runs (see Methods for details). Fig. 2 demonstrates an example run of the CVFS approach (see Methods for more explanations and details of this figure). We tested several settings, including the number of distinct sub-parts (i.e. the *n* split number mentioned in Algorithm) and the frequency of observing selected genes from repeated runs (i.e. the *Z*% minimum observation frequency cutoff among all *R* repeated runs described in Algorithm 1), on both bacterial pan-genome. The results are shown in Supplementary Fig. S1, which indicate that applying XGBoost as the feature selection algorithm along with “*n* = 2″ and “*Z* = 80%” settings yields good performances with relatively low feature number. We thus proceeded with this setting for the ongoing analysis.

**Fig. 3:**
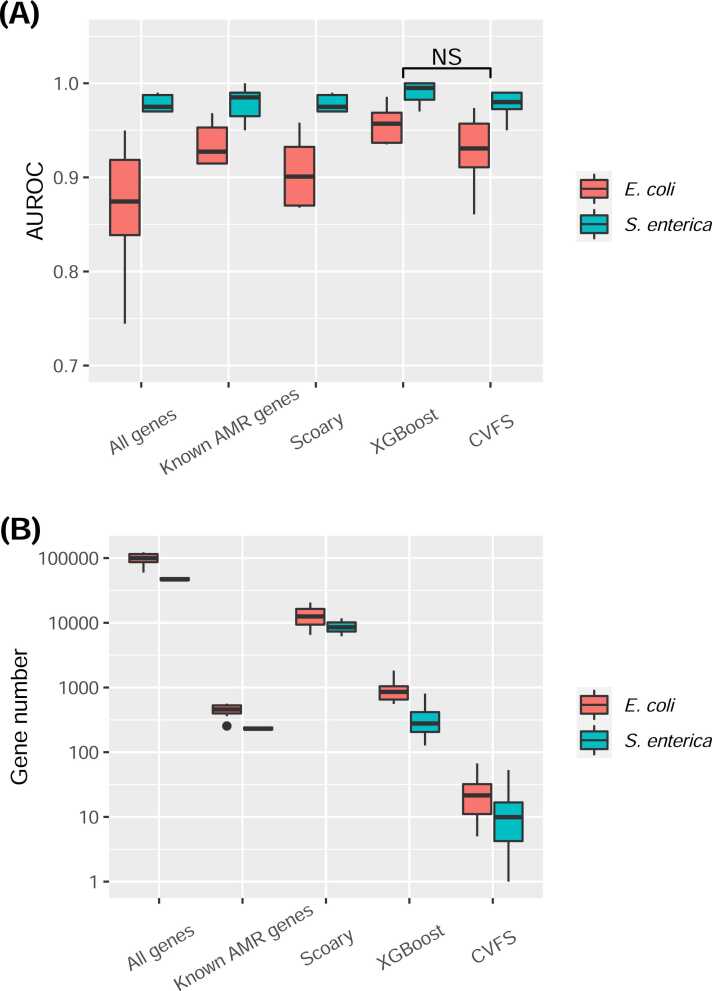
The evaluation results of different gene sets from two bacteria. The results include (A) prediction performance in terms of Area Under Receiver Operating Characteristic curve (AUROC), and (B) the number of genes in different gene sets. X-axis indicates different gene sets (see text for details), and Y-axes in (A) and (B) are AUROC and gene numbers, respectively. The AUROC evaluation was conducted by training SVM model on different gene sets and evaluated the model performances using stratified ten-fold cross validation. The Y-axis in (B) is log(10)-scaled in order to take into account of the huge differences between the number of genes in different gene sets. “NS” indicates that the differences are not statistically significant.

The antimicrobial resistance phenotypes of the antibiotic drugs were integrated with the gene presence/absence patterns of the pan-genome data in order to build the prediction models. Ten drugs for each of the bacterial species satisfied our selection criteria (See Methods and Supplementary Table S1 for details) and were included into the analysis. The performance of the CVFS approach was compared against other settings, including 1) using all genes, 2) using known antimicrobial resistance genes (predicted using CARD/RGI; see Methods), 3) using Scoary (with default settings), a rapid gene scoring system for microbial pan-genome-wide association [7], and 4) using genes selected by applying feature selection algorithm (XGBoost in this case) on the entire dataset. The results clearly showed that pan-genome was a very effective tool for conducting AMR prediction. As shown in Fig. 3(A), the prediction power of the CVFS algorithm was comparable to other gene sets, including all genes, known AMR genes, Scoary-selected genes, and XGBoost-selected genes (Wilcoxon rank-sum test; p = 0.16, 0.63, 0.22, and 0.28 respectively). The numbers of genes extracted by the CVFS algorithm, however, were significantly lower than other gene sets (Wilcoxon rank-sum test; all p-values<<0.001), as shown in Fig. 3(B) (detailed numbers for plotting Fig. 3 were provided in Supplementary Table S2-S5). The significantly lowered gene numbers indicated that the CVFS algorithm was able to extract the most parsimonious feature sets with good prediction performances and hinted that the CVFS-genes may perhaps be more relevant to the AMR mechanisms.

### 2.3 Functional analysis of selected genes

To understand the functional aspects of the CVFS-genes selected by the cross-validated feature selection approach, we analyzed the distribution of functional annotations of the genes (Supplementary Table S6). Not surprisingly, we found some well-known AMR genes among the genes identified by the CVFS approach (Supplementary Table S7). For example, beta-lactamase genes including *bla*_CMY-2_, *bla*_CARB-3_, *bla*_TEM-1_, and *bla*_KPC-2_ were identified for beta-lactam antibiotics. Similar observations were also made on other drugs, including *tetA* and *tetB* for tetracycline resistance, *sul1* and *sul2* for sulfisoxazole resistance, *cmlA1* and *floR* for chloramphenicol resistance, and *dfrA1*, dfrA8, *dfrA14*, and *dfrA17* for trimethoprim resistance, etc. This finding indicated that the CVFS approach was capable of identifying known AMR genes that were relevant to the AMR mechanisms.

**Fig. 4:**
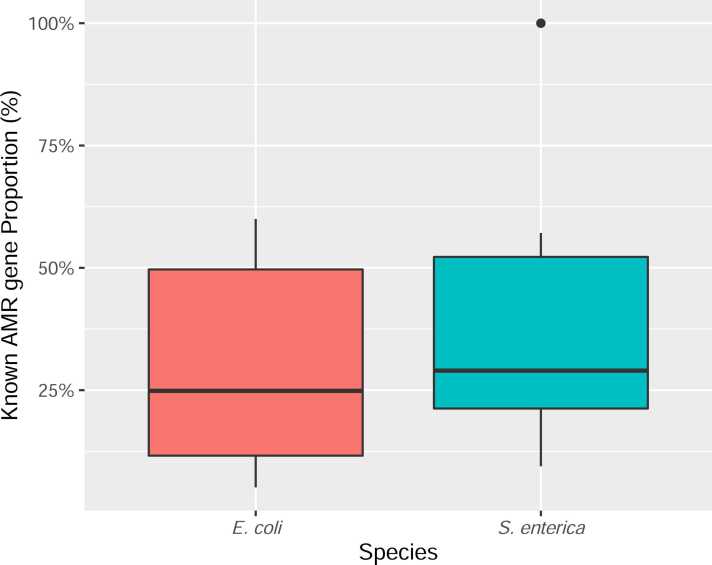
Distribution of the proportion of known AMR genes among genes identified by the CVFS approach.

**Fig. 5:**
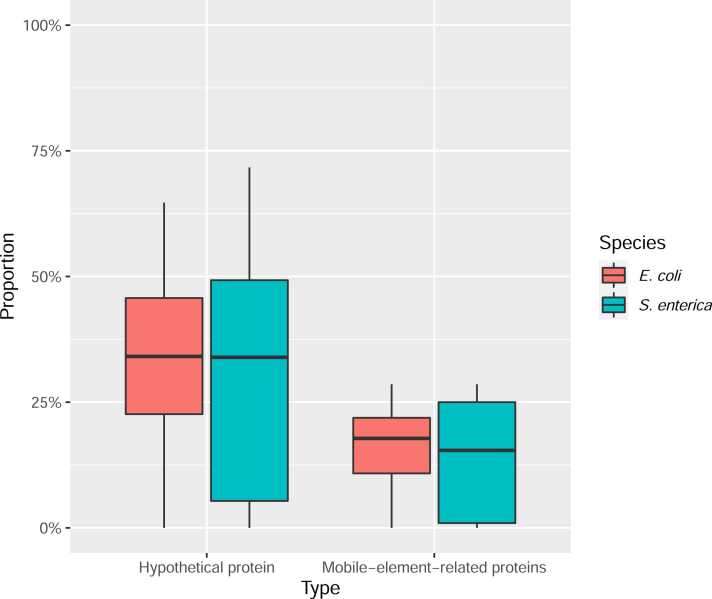
Distribution of proportions of genes annotated as hypothetical proteins and related to mobile elements among genes identified by the CVFS approach.

However, not all CVFS-genes belonged to the genre of known AMR genes, as the proportion of AMR genes accounted for less than 50% for most of the drug datasets (Fig. 4). Meanwhile we found that a significant proportion of the identified CVFS genes were functionally-unknown. As shown in Fig. 5, the mean proportion of hypothetical proteins (i.e. genes with unknown functional roles) was around 30% (detailed hypothetical protein proportions were provided in Supplementary Table S8 and S9), indicating the high association of those hypothetical proteins with the AMR phenotypes and hinting that some of the functionally-unknown genes may play uncharacterized roles in AMR mechanisms.

Besides hypothetical proteins, the next most annotated functional type was mobile elements- or phage-related genes. The average numbers of genes related to mobile elements were around 15–20% (Fig. 5; see Supplementary Table S8 and S9 for detailed numbers), indicating genes related to either transporting AMR genes between different bacterial cells or uptaking AMR genes from the surrounding environments were also significantly associated with AMR activities.

## 3 Discussion

In this paper we proposed a cross-validated feature selection approach for extracting the most parsimonious AMR gene sets from bacterial pan-genomes. By testing this approach on the AMR datasets of two bacterial species, we showed that the number of CVFS-genes were indeed much fewer than either contemporary machine learning feature selection approaches or known AMR gene sets extracted using database-based methods, indicating that the CVFS-genes may be more significantly associated with AMR. For example, in the *Salmonella enterica* dataset, 98% prediction accuracy can be achieved using only two CVFS-genes to build the machine learning prediction model for predicting amoxillin/clavulanic acid drug resistances. We note that even though applying XGBoost algorithm on the entire *S. enterica* amoxillin/clavulanic acid dataset yields 127 genes and achieves 100% prediction accuracy, and that using 231 known AMR genes achieves 99% prediction accuracy, the number 127 and 231 are significantly higher than just two CVFS-genes for achieving 98% accuracy, indicating the worthiness of these two genes to be further explored in studying *S. enterica* amoxillin/clauvulanic acid resistance mechanisms due to their high prediction capabilities.

The model performance evaluations demonstrated that XGBoost-selected genes (without CVFS) perform best among all gene sets, as shown in Fig. 3(A). One may then ask: why bother employing the CVFS idea instead of just applying XGBoost on the entire dataset? We argue that there are two reasons for applying CVFS. Firstly, even though the XGBoost-selected genes performed very well, the number of genes extracted by the XGBoost algorithm still reached the scale of several hundred; meanwhile the numbers of CVFS-genes were all less than 100 (the average number of genes for *S. enterica* and *E. coli* were 14 and 10.5 respectively) with very similar prediction performances for all conducted tests. In other words, the far fewer CVFS-gene numbers along with comparable resistance prediction performances make them much more worthwhile in ongoing exploration of AMR mechanisms or serving as AMR biomarkers. Secondly, since the CVFS-genes were extracted by repeatedly and independently interrogating the data, they may be more representative and more strongly-correlated to the AMR phenotypes. Although the possibility of overfitting cannot be completely ruled out and is difficult to be measured quantitatively, we emphasize that the design of CVFS is to ensure the representativeness of features from distinct sub-parts, which may partly mitigate overfitting since the features are extracted and “cross-validated” from distinct sub-parts. The comparable classification performances of CVFS-genes with other feature selection and classification methods also strongly support the representativeness of the CVFS-genes. We also compared the CVFS algorithm against several other feature selection methods, including random forest, Lasso, and recursive feature elimination with cross validation (RFECV) conducted on SVM. The results, as shown in Supplementary Fig. S2, also showed that the CVFS algorithm is superior to other algorithms, in which the CVFS approach was capable of extracting the lowest amount of features and achieving comparable classification performances.

We also note that the CVFS approach does not belong entirely to the feature-importance-ranking-filtering category among feature selection algorithms (including but not limited to tree-based algorithms such as decision tree or random forest or some of the wrapper models such as forward selection or backward elimination methods that rely on feature importance) since the CVFS approach does not consider feature importance scores; instead it looks for common features between different sub-parts (randomly-generated disjoint sub-tables) and among repeated runs. One of the evidences is that the features extracted by the CVFS approach do not correspond solely to the top-ranking features. For instances, among the five genes picked by the CVFS approach for *S. enterica* cefoxitin resistance, only one gene ranked first among the 226 genes selected and ranked by the XGBoost algorithm; other genes were ranked much lower (lower than 100th rank). Similarly, among the two *S. enterica* genes extracted for the amoxicillin/clavulanic acid resistance, only one ranked first among those ranked by XGBoost; the other gene was ranked much lower. In other words, the CVFS approach goes beyond the importance ranks and is capable of finding features for consistently predicting AMR activities without the influences of importance scores or ranking information.

In a quick glance this approach may look similar to the bagging (bootstrap aggregation) approach [23], which is an ensemble learning algorithm that aims to sample (with replacement) from the learning space to generate multiple weak learners and aggregate their prediction power to build a strong learner. We however note that the CVFS approach is different from the bagging approach in at least two ways. Firstly, instead of prediction accuracy, the CVFS approach puts more emphasis on extracting the features (in this case gene clusters) that are clearly associated with the outcomes (for example, AMR phenotypes). Secondly, in contrast to the sampling-with-replacement approach conducted by bagging, the CVFS approach takes non-overlapping subsets of features in order to achieve its “cross validation” goal, in which features extracted from different subsets are intersected to identify the most representative feature set for associating and predicting the AMR outcomes. We thus acknowledge the similarities in the weak learner idea but would like to stress the differences between the goals and approaches of the bagging and CVFS approach.

The experimental evaluation was conducted on drugs with enough data entries (at least 900 records) and at most 1:10 ratio between R:S or S:R phenotype proportions. To test the influences of data entry sizes, we tried to conduct the analysis on three smaller *S. enterica* drug datasets (spectinomycin, trimethoprim, and sulphonamides), in which the amount of data associated with the three drugs were significantly lower but still within 1:10 R-S ratio (resistant and susceptible strains for the three drugs are 29:247, 39:291, and 270:31, respectively). As shown in Supplementary Table S10, even though the spectinomycin and trimethoprim datasets still yielded 93% and 94% accuracies and outperformed both “all genes” and “XGBoost” feature selection approaches, the performance of sulphonamides dataset was significantly lower (66%). This indicates that enough data entries may be needed for conducting the CVFS approach successfully.

We also tested how data balance affects the extraction of CVFS-genes. As was also shown in Supplementary Table S10, the application of CVFS approach on two *S. enterica* drug dataset (nalidixic acid and kanamycin) with more-than-ten-fold balance degree (R:S=1:16 for both drugs) still yielded some results; however the prediction performances (81% and 89%) were lower than other datasets with more balanced data distribution. This result suggests that more balanced data distribution, along with enough data entries, are perhaps necessary for the CVFS approach to be fully functional. In our future work we plan to devise plans to determine how many data entries or what properties of data (e.g. class balance degrees) warrants application of the CVFS approach on any given dataset.

We note that despite the capability of identifying much fewer genes for achieving comparable performances with XGBoost or random forest, the CVFS approach takes longer time to run. By using *S. enterica* data to evaluate the time required to run different feature selection algorithms, we found that the average amount of time running CVFS, XGBoost, and random forest feature selection was 640, 221, and 7 seconds respectively (tested on a Ubuntu linux 16.04 xenial server using four Intel Xeon ES-2630 2.20 GHz cores). This result was not surprising since the core idea of CVFS was the repeated splitting of the dataset and the execution of feature selection on each subset of the input data. We however emphasize that the time required for running CVFS is still acceptable (about 10 min for running a dataset with thousands of features and samples), and that the ability of extracting much fewer features with similar prediction power is more important than running time alone.

From the functional annotation analyses we found that a significant amount of CVFS-genes were hypothetical proteins, indicating that they are functionally-unknown. Since the CVFS-genes were intersected from the features selected using XGBoost algorithm for each randomly-splitted sub-table, we hypothesized that significant proportion of genes identified by XGBoost may be hypothetical proteins. Indeed, more than 50% of XGBoost-selected *S. enterica* genes were of unknown function as shown in Supplementary Table S11. We also observed that about 10% of genes were related to mobile-element proteins, similar to the CVFS-gene distribution. A previous study also made very similar discoveries [22]. As suggested in Results, the genes with unknown functions may worth further exploration due to their significant associations with AMR phenotypes. We again emphasize that even though the XGBoost-selected genes (without CVFS approach) can be used to build the prediction model with the highest prediction accuracy, the numbers of CVFS-genes were far fewer than the XGBoost-selected genes (p<<0.001) but were able to achieve very similar prediction performance (p > 0.1), indicating the CVFS-genes merit more attention in looking for reasons for their associations with drug resistances. We plan to apply methodologies such as the PangenomeNet [24] to infer the functional roles of the hypothetical proteins in our future work.

By comparing the CVFS-genes against known AMR genes, we found that only a portion (ranged from 6% to 100%) of CVFS-genes can be identified in existing AMR databases. We again went back to check the XGBoost-selected genes and found that less than 6% (from 0.88% to 5.51%) were known AMR genes (Supplementary Table S12), as was also observed in [22]. Such partial overlap with known AMR genes may indicate two things: 1) genes with known AMR functional roles may only contribute partly to AMR activities, and 2) some functionally-uncharacterized genes may also play some roles for the bacterial species to withstand antibiotic drugs. We also identified well-established genes associated with corresponding AMR mechanisms, indicating that the CVFS approach was able to identify both known and unknown genes associated with AMR activities.

Since the gene functional annotations were provided by the PATRIC database, it is possible that genes annotated as “hypothetical proteins” may actually be known AMR genes due to outdated annotations. We conducted a double check on the hypothetical proteins but did not observe any of the functionally-uncharacterized genes that can be annotated as known AMR genes by the CARD/RGI database. We of course did not rule out the possibility that some of the hypothetical proteins may be annotated otherwise by searching against some other databases; however for our purpose, which was looking for AMR genes, we did not find any hypothetical proteins that were actually known AMR genes. In our future work we plan to continually utilizing other databases to confirm the annotations and attempting to look for hints about why some of the functionally-unknown genes were highly-associated with AMR.

Despite the ability of the CVFS approach to extract highly associated features from the resistance dataset in an unbiased manner, there are still limitations of this approach. The first limitation is related to the dataset size and balance. In this study we intentionally selected drug resistance data with more than 900 entries and less than ten-fold data coverage difference. However at current stage we cannot say for certain how many data entries or how balanced is “enough” to warrant the application of the CVFS method. The second limitation is about the data nature. In this study we applied CVFS on the pan-genome data, which was transformed into gene presence (1) and absence (0) pattern for each of the genomes. We however are not sure whether this method can be extended to other types of data, for example numerical data or nominal data with three or more possible labels. These two limitations need to be further explored in order to extend this methodology to other applications.

Another limitation is that SNPs (single nucleotide polymorphism) or allele variations are not included in this study due to the gene-clustering nature of pan-genomes, in which genes with above-threshold identities are grouped into the same cluster. We however note that even if SNPs were not included into our analysis procedure, we were still able to build models with very good predictive ability by extracting the most parsimonious feature sets. Hence one can think of our approach as accessing the AMR problem in the perspective of the presence or absence of gene clusters or families, and that we were attempting to look for the most informative genes/gene clusters for determining and predicting AMR activities. At the same time we do not intend to overlook the importance of SNPs for the occurrence of AMR, as several studies have identified important SNPs for AMR phenotypes (e.g. [25], [26]). We plan to incorporate SNPs/allele-variations together with gene families for AMR prediction in our future work.

Lastly, even if the CVFS approach is able to extract as few features as possible for predicting AMR activities, the high prediction accuracies do not automatically translate to AMR mechanisms. As a result we used terms such as “genes highly-associated with AMR activities” or “genes related to AMR” to indicate that these genes are correlated with AMR and can be incorporated into machine learning models for prediction purpose; however we cannot say for sure these genes or their protein products contribute to the AMR mechanisms. This is especially critical since we observed a huge proportion of genes with unknown functions that we cannot say anything about whether or how they influence AMR phenotypes. We would also like to point out that the CVFS approach may help in this aspect, in which the most parsimonious gene sets extracted unbiasedly from cross-validated and repeated procedure may represent highly-associated genes in a better way, and that the much reduced gene sets may serve as guidance in designing experimental validations (discussed in the next paragraph). In other words, the superiority of features (genes) extracted by the CVFS approach lies in both the low number of features and the representative nature of the features in associating and predicting AMR outcomes.

Since the primary goal of CVFS is to select the most parsimonious feature set while still maintaining high prediction performances, the selected feature sets may serve as guidance in further research works. There are several important fronts that may benefit from the parsimonious feature sets selected by the CVFS approach. The first research front would of course be the discovery of novel AMR genes, in which CVFS are able to extract functionally-uncharacterized genes that are highly-associated with resistance phenotypes. These genes may be further explored to check whether they are really functionally related to resistance mechanisms using experimental validations or computational methods such as Alphafold2 [27]. Other research fields may also benefit from the CVFS approach, for example the extraction of pan-cancer molecular biomarker [28], the identification of gene biomarkers for Mendelian disorders [28], [29], or gene signature for drug repositioning [30]. Therefore the capability of selecting the most parsimonious and representative feature sets using the CVFS approach may help in many research aspects, especially those in need of marker or signature discoveries. We also foresee that the identification of using and selecting as fewer genes as possible may be beneficial to the detection of AMR strains or other molecular diagnostics using gold-standard PCR tests, as was also discussed in [31], and the capability of CVFS can be very helpful in this aspect.

## 4 Materials and methods

In this section we describe the methodology details in building the pan-genome, selecting the AMR data for analysis, and the steps for conducting cross-validated feature selection and evaluation.

### 4.1 Pan-genome construction

The *Escherichia coli* and *Salmonella enterica* genomes and genes (including.fna and.faa files) and the antimicrobial resistance phenotype metadata (PATRIC_genomes_AMR.txt) were downloaded from the PATRIC database [20] (https://www.bv-brc.org/). Prior to the construction of the pan-genomes, we first checked the genome qualities and potential contaminations. The genome qualities were checked using CheckM v1.1.3 [32], and the contaminations were identified by downloading the 16 S ribosomal RNA subunit gene from NCBI-listed *E. coli* and *S. enterica* reference genome (*E. coli* K-12 substr. MG1655 and *S. enterica* subsp. enterica serovar Typhimurium str. LT2; NCBI acc. NC000913 and NC_003197) and querying it against all downloaded genomes using BLASTN [33]. Only genomes with 1) CheckM completeness> 95% and contamination< 5%; and 2) BLAST 16 S nucleotide identity> 99% were included into the pan-genomes. The construction of the pan-genomes was then conducted by collecting the genes in amino acid sequences into a file, one for each of the species, and clustering the sequences using CD-HIT v4.6 [34] at 95% amino acid identity following [21]. The pan-genome rarefaction distribution was estimated by randomly sampling the genomes and cumulatively calculating the numbers of core and accessory genes, in which core and accessory genes were defined according to whether genes exist in all (100%) of the genomes or not, respectively. The random sampling process was repeated 10 times to get the averaged cumulative gene count values. Known AMR genes were identified by applying the Resistant Gene Identifier (RGI) software v5.1.1 [35] provided by CARD on the CD-HIT representative gene sequences.

The determination of whether the pan-genomes belong to an open-genome or a close-pan-genome was performed following [36], [37]. Briefly the pan-genome growth curve was fit to a power law growth model $n=\kappa N^{\gamma }$, where *n* was the averaged pan-genome size from the 10 random sampling process and *N* is the number of genomes (note that averages and medians of the pan-genome sizes from the random sampling process yielded consistent power law growth model component estimates according to [36]). If the exponent γ>0, the pan-genome was then determined to be an open-pan-genome [36].

### 4.2 Associating pan-genome and AMR activities

The antimicrobial resistance metadata downloaded from PATRIC was processed to identify the “Resistant” and “Susceptible” labels in the “Resistant Phenotype” field, in which resistant indicated that the specific bacterial strain was resistant to the corresponding antibiotic drug and susceptible otherwise. We also made sure that evidences of the resistant phenotypes were based on laboratory methods or were conducted using wet-lab laboratory typing methods (i.e. no computationally annotated phenotypes were included in the analysis) by cross-comparing the resistance metadata using the PATRIC website. Other labels such as “Intermediate” or “Not defined” accounted for only 0.5% of the resistance phenotypes and were thus excluded from the analysis. Only antibiotic drugs with enough resistance phenotype annotation data were included in the analysis, in which the criteria were: 1) at least 900 phenotype data entries for the combination of resistant (R) and susceptible (S) labels, and 2) the maximum ratio between R and S was ten-fold or less (indicating that the data should not be too unbalanced). The pan-genome content was then associated with the AMR phenotypes by indicating whether gene clusters were present (1) or absent (0) for the analyzed genomes.

### 4.3 Cross-validated feature selection

In general, the cross-validated feature selection algorithm starts by splitting the pan-genome data into *n* sub-parts, extracting the features (that is, genes) highly associated with the phenotypes using eXtreme Gradient Boosting (XGBoost) algorithm [38], and intersecting the identified features in order to find common ones that appear in all distinct sub-parts (see an example in Fig. 2(A)). This randomized-splitting/feature selection/feature intersection process is then repeated several times to identify features that are repeatedly discovered in the majority (at least *Z*%) of runs (Fig. 2(B)). The algorithm was given in Algorithm 1, and the detailed procedure was described as follows.

Algorithm 1 Cross-Validated Feature Selection.

**Table:** 

The first step of the algorithm is stratified splitting, in which each of the pan-genome-AMR association tables is split into *n* sub-parts while preserving the proportion of R and S. For example, let us assume that there are 1000 genomes (300 resistant and 700 susceptible) in the pan-genome with 20,000 gene clusters. If we are to split the pan-genome tables into two sub-tables (i.e. *n* = 2), the splitting results are going to be two table, each consists of 500 genomes (150 resistant and 350 susceptible) and 20,000 gene clusters.

After separating the pan-genome tables into sub-tables, the next step is to employ XGBoost to select features that are most relevant to the phenotypes from each of the sub-tables. The feature selection procedure was conducted by applying XGBoost binary classifier (objective=binary:logistic with default parameters, in which number of estimators=100, maximum tree depth=6, and importance type=gain; the hyperparameter setting was supported by a grid search on XGBoost parameters using *S. enterica* datasets shown in Supplementary Fig. S3) on the sub-tables and extracting the features used to build the XGBoost trees (all features are extracted regardless of their gain scores). After the features, which are gene clusters from the pan-genome, are extracted from each individual sub-table, they are then intersected to find common features among all sub-tables (i.e. XGBoost-selected features shared by different sub-tables). This process is independently repeated *R* times (*R*=5) in order to find features that are consistently uncovered in most of the runs.

After the features are extracted from the dataset, the performance of the features is estimated by building the Support Vector Machine (SVM) machine learning model (linear kernel with default C=1.0); the model is then evaluated using stratified ten-fold cross-validation. The independent variables are the extracted features (that is, gene presence/absence patterns identified from the bacterial pan-genomes) while the dependent variables are the resistance phenotypes against the antibiotic drugs (i.e. resistant (R) or susceptible (S)). The use of linear SVM as the classification model was based on the comparison of different classification algorithms (Supplementary Fig. S4) and SVM hyperparameter grid search results (Supplementary Fig. S5), which showed that the SVM (which was least affected by random effect compared to other algorithms) with a simple linear kernel and default hyperparameter settings was adequate to serve as classifier. The Area Under the Receiver Operating Characteristic curve (AUROC) was selected as the evaluation metric to compare the performance between different gene sets. The machine learning implementation was conducted using the Scikit-learn [39] (https://scikit-learn.org/) and XGBoost Python packages (https://xgboost.readthedocs.io/).

### 4.4 Functional analysis of the selected genes

The functional annotation of the CVFS-genes was taken directly from existing PATRIC annotation data. Genes with “hypothetical” terms in its annotations and were not annotated by CARD/RGI as known AMR genes were regarded as unknown functional roles, and genes with terms “mobile,” “phage,” “transposase,” “integrase,” or “tail fiber assembly” were classified into mobile-element-related proteins.

## Author statement

MRY conducted the analysis and participated in draft writing; YWY conceived the study, conducted part of the analysis, and wrote the manuscript.

## Conflict of interest

The authors have no conflicts of interest to declare. All co-authors have seen and agree with the contents of the manuscript and there is no financial interest to report. We certify that the submission is original work and is not under review at any other publication.

## Data Availability

The source code of the cross-validated feature selection approach and the pan-genome gene presence/absence data are available at the Github repository https://github.com/mingren0130/CVFS_code.
