# Supplementary material for: A Cross-Validated Feature Selection (CVFS) approach for extracting the most parsimonious feature sets and discovering potential antimicrobial resistance (AMR) biomarkers
Source: Comput Struct Biotechnol J. 2022 Dec 28;21:769–79. doi: 10.1016/j.csbj.2022.12.046 (PMC9842539; doi:10.1016/j.csbj.2022.12.046)
Supplement: Supplementary file 1 — Supplementary material [file mmc1.pdf]

A Cross-Validated Feature Selection approach for extracting the most parsimonious feature sets and discovering potential antimicrobial resistance (AMR) biomarkers

Ming-Ren Yang and Yu-Wei Wu

Supplementary materials:

Supplementary Figures S1-S5

Supplementary Tables S1-S12

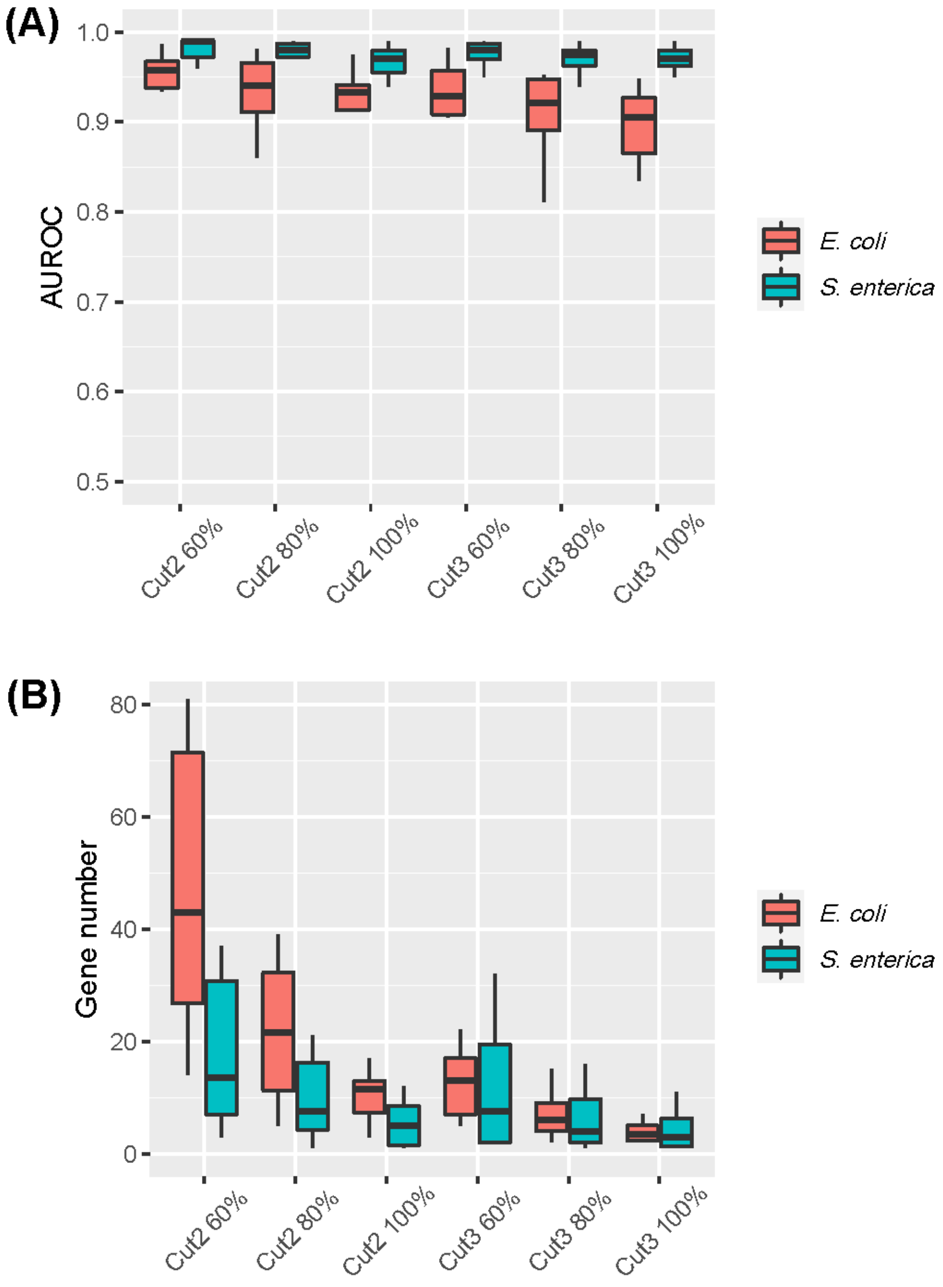

Supplementary Figure S1. Accuracy (in terms of area under receiver operating characteristics curve, or AUROC) and the number of genes for different CVFS algorithm settings. "Cut2" or "Cut3" indicates the number of distinct sub-tables split from the main table (the "*n*" parameter in Figure 2 of the manuscript), and the percentages represents the frequency of observing selected genes from repeated runs (the "*Z%*" parameter in Figure 2 of the manuscript).

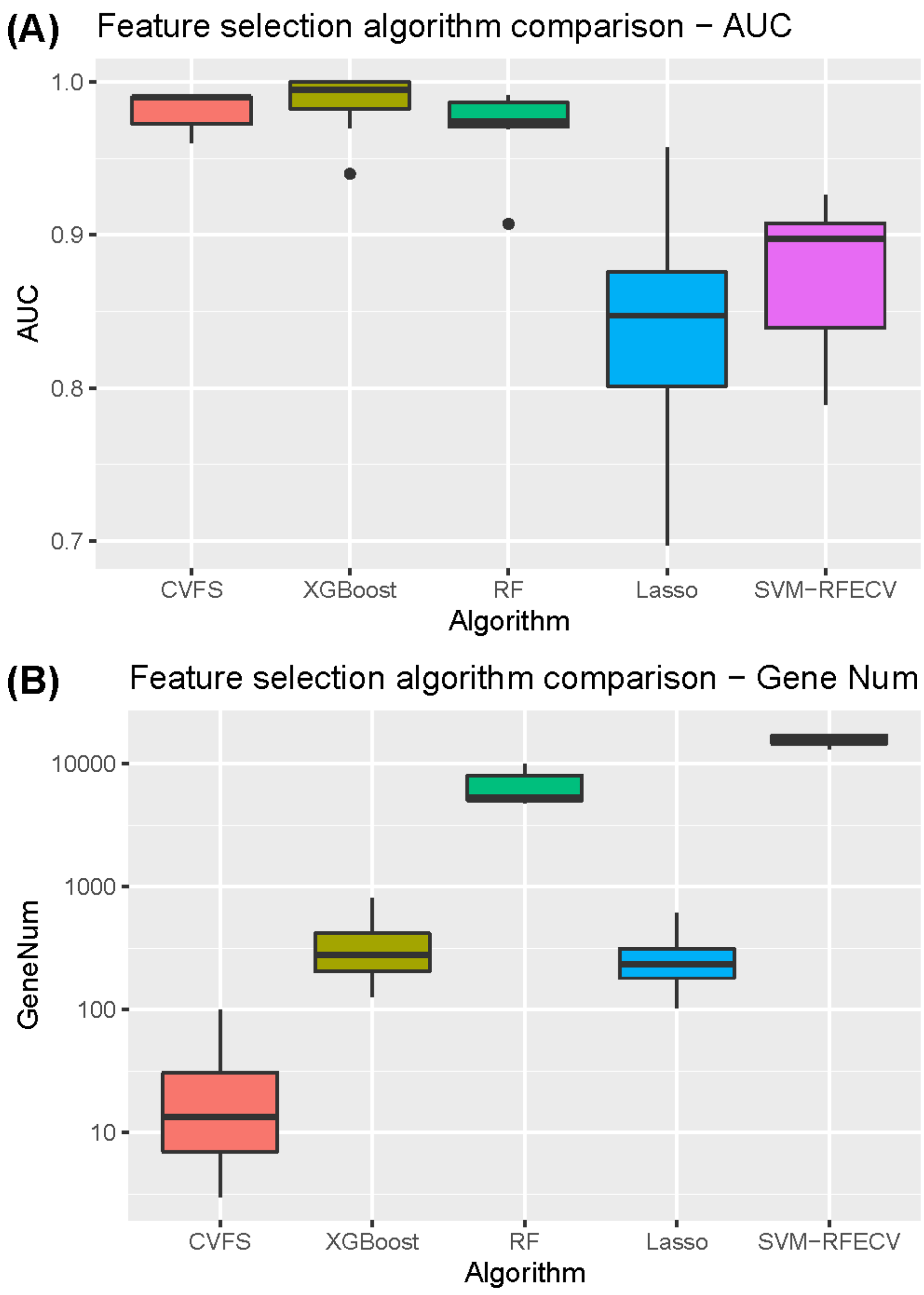

Supplementary Figure S2. Classification accuracies and gene numbers (in terms of area under receiver operating characteristics curve, or AUROC) of the *S. enterica* datasets measured for additional feature selection algorithms, in which the feature selection was conducted using different methods followed by linear SVM classification (C=1). The CVFS and XGBoost results were the same as shown in Figure 2. The random forest feature selection was conducted by selecting features with importance score > 0. The Lasso algorithm was run using default parameter setting (alpha=1), and the SVM-RFECV (stands for Support Vector Machine feature selection with linear kernel and default settings using

Recursive Feature Elimination Cross Validation) was run using Scikit-learn package with elimination step set as 1000 due to the vast amount of features.

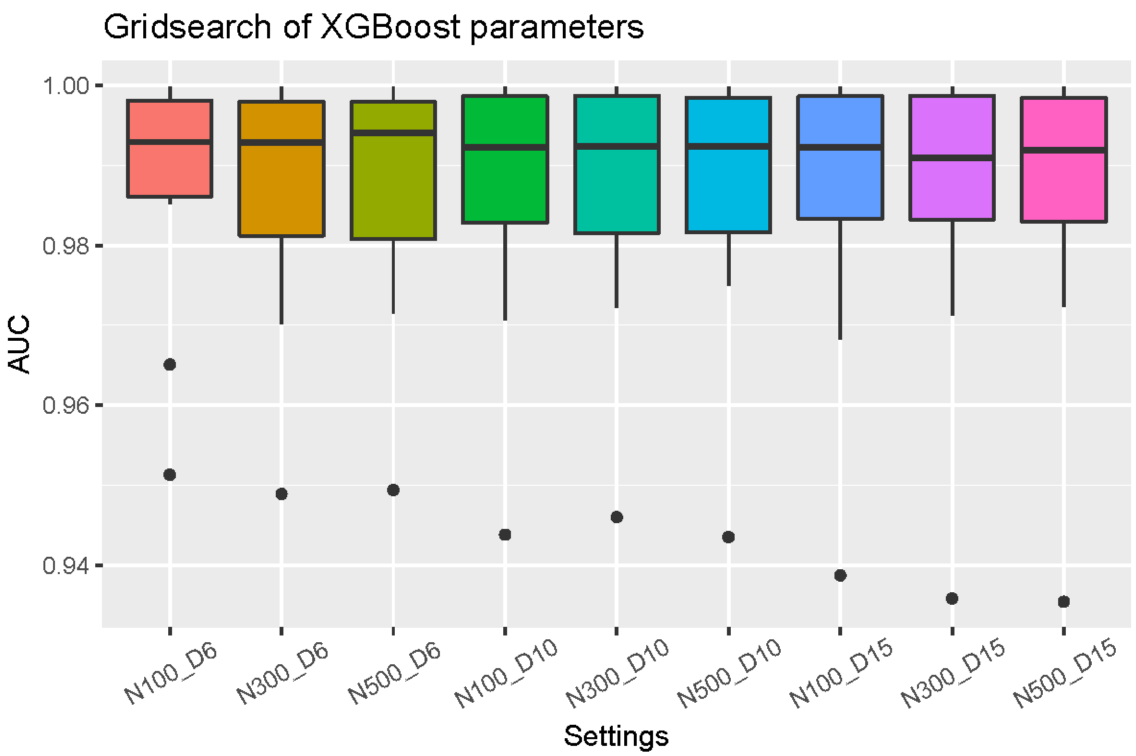

Supplementary Figure S3. Hyperparameter grid search of XGBoost parameters on the *S.enterica* datasets. N stands for the number of boosting rounds (n_estimators) and D is the maximum tree depth (max_depth). The default setting of XGBoost is N100_D6, which is also the setting incorporated in CVFS base algorithm. All results are not statistically different from each other (Wilcoxon rank sum test P > 0.5 for all pairs of results)

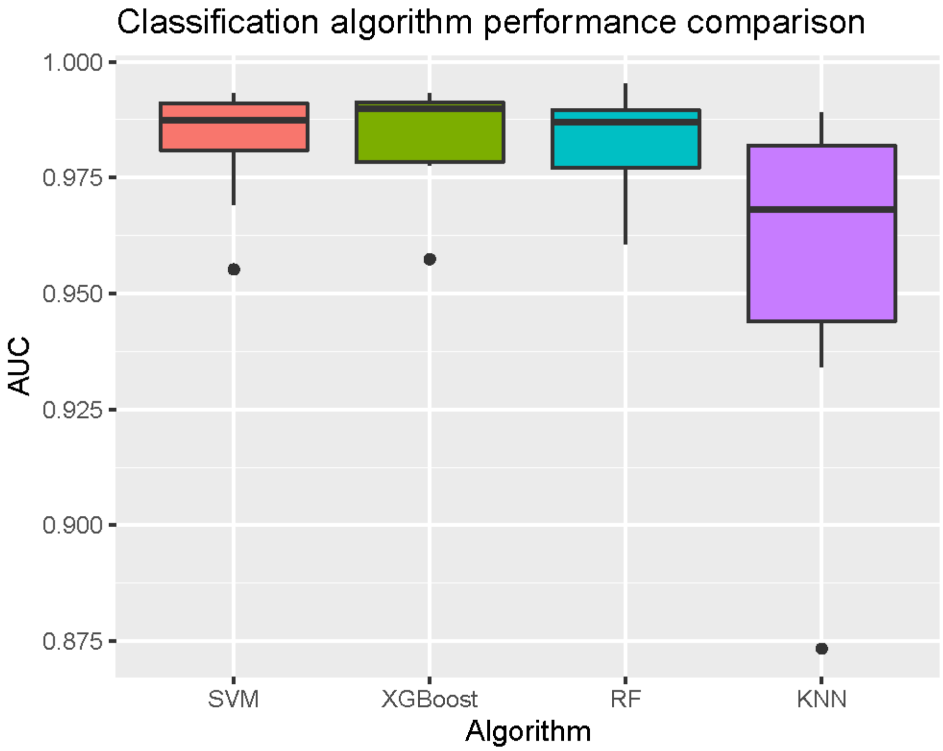

Supplementary Figure S4. Performance comparisons of different classification algorithms conducted on the CVFS-selected features of *S. enterica* datasets. The settings were: SVM with linear kernel and default settings (C=1); XGBoost with max_depth=6 and boosting round=100; random forest (RF) with 100 trees; and KNN with k=5. The differences between SVM, XGBoost and random forest were not statistically significant (Wilcoxon rank sum test P > 0.5 for all pairs of results except KNN).

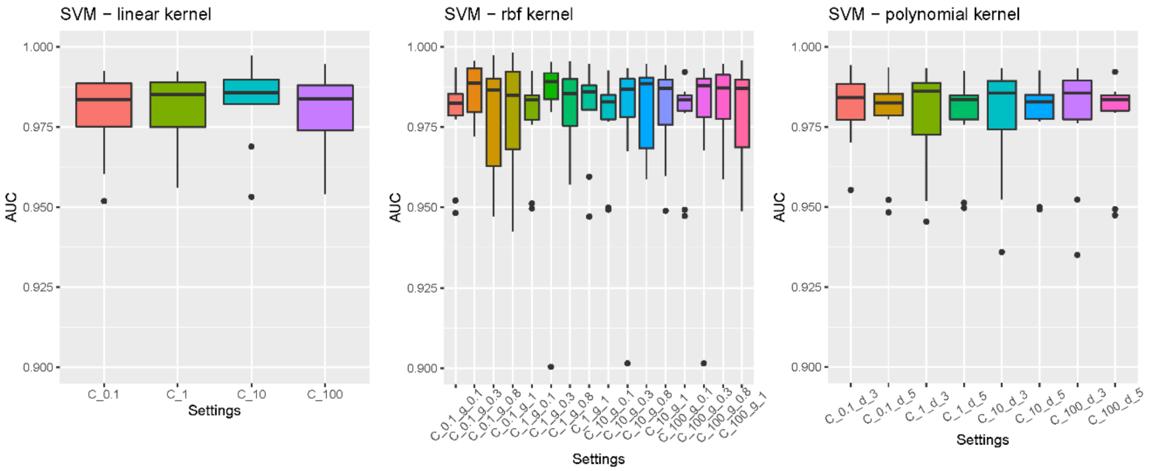

Supplementary Figure S5. Performance comparisons for different support vector machine (SVM) kernels and settings. The “C” in all three kernels stands for the regularization parameters. The “g” in rbf kernel stands for gamma, and the “d” in polynomial kernel stands for degree. No significant differences are observed for all pairs of results (Wilcoxon rank sum test P > 0.1 for all possible pairs of results from all three kernel settings).

Supplementary Table S1. The numbers of resistant and susceptible strains for the antibiotic drugs. Blue drug names indicate drugs included in the analysis of this study.

	Antibiotic drug	Susceptible (S)	Resistant (R)	R+S
S. enterica	Ampicillin	1242	906	2148
	Streptomycin	934	1193	2127
	Tetracycline	948	1178	2126
	Chloramphenicol	1720	402	2122
	Gentamicin	1748	195	1943
	Sulfisoxazole	629	1168	1797
	Ceftriaxone	1432	345	1777
	Ceftiofur	1429	338	1767
	Cefoxitin	1462	279	1741
	Amoxicillin/Clavulanic acid	1402	338	1740
E. coli	Ciprofloxacin	1718	855	2573
	Ceftazidime	1914	650	2564
	Gentamicin	2099	441	2540
	Amoxicillin/Clavulanic acid	1474	744	2218
	Cefotaxime	1513	653	2166
	Ampicillin	432	1542	1974
	Cefuroxime	1403	489	1892
	Amoxicillin	466	713	1179
	Tobramycin	823	308	1131
	Trimethoprim	507	475	982

Supplementary Table S2. SVM prediction accuracies (in terms of AUROC) using the gene clusters selected by different approaches for the drug resistance profiles of *Salmonella enterica*.

	All genes	CARD	XGB[1]	Scoary	CVFS
Ampicillin	0.97	0.98	0.98	0.97	0.98
Streptomycin	0.90	0.95	0.94	0.78	0.95
Tetracycline	0.97	0.98	0.99	0.97	0.98
Chloramphenicol	0.98	0.99	0.99	0.98	0.99
Gentamicin	0.97	0.96	0.97	0.97	0.95
Sulfisoxazole	0.99	0.99	1.00	0.99	0.99
Ceftriaxone	0.99	1.00	1.00	0.99	0.99
Ceftiofur	0.99	0.99	1.00	0.99	0.97
Amoxicillin/Clavulanic acid	0.97	0.99	1.00	0.97	0.98
Cefoxitin	0.98	0.95	1.00	0.98	0.99

[1]XGB stands for XGBoost

Supplementary Table S3. SVM prediction accuracies (in terms of AUROC) using the gene clusters selected by different approaches for the drug resistance profiles of *Escherichia coli*.

	All genes	CARD	XGB[1]	Scoary	CVFS
Ciprofloxacin	0.94	0.91	0.97	0.94	0.97
Ceftazidime	0.92	0.93	0.96	0.93	0.97
Gentamicin	0.91	0.95	0.98	0.93	0.93
Amoxicillin/Clavulanic acid	0.68	0.82	0.81	0.72	0.82
Cefotaxime	0.95	0.97	0.99	0.96	0.98
Ampicillin	0.89	0.95	0.96	0.91	0.96
Cefuroxime	0.74	0.78	0.84	0.76	0.86
Amoxicillin	0.83	0.92	0.94	0.87	0.91
Tobramycin	0.86	0.92	0.95	0.89	0.91
Trimethoprim	0.85	0.95	0.94	0.88	0.95

[1]XGB stands for XGBoost

Supplementary Table S4. Numbers of gene clusters selected by different approaches for *Salmonella enterica*.

Antibiotic drug	All genes[1]	CARD[2]	XGB[3]	Scoary	CVFS
Ampicillin	49539	248	486	11240	21
Streptomycin	49130	241	804	10207	53
Tetracycline	49149	245	442	11746	17
Chloramphenicol	49123	241	348	9496	14
Gentamicin	47652	227	320	6191	7
Sulfisoxazole	45738	223	241	9949	16
Ceftriaxone	45506	229	192	7369	4
Ceftiofur	45399	222	199	7312	1
Amoxicillin/Clavulanic acid	45874	231	127	7672	2
Cefoxitin	45359	222	226	6311	5

[1]The number of gene clusters after excluding all-zero gene clusters from the drug presence and absence table (i.e. gene clusters not present in any of the strains for this drug).

[2]Known AMR gene clusters predicted by CARD after excluding all-zero gene clusters from the drug presence and absence table.

[3]Results yielded by applying XGBoost feature selection algorithm on the drug datasets.

Supplementary Table S5. Numbers of gene clusters selected by different approaches for *Escherichia coli*.

Antibiotic drug	All genes[1]	CARD[2]	XGB[3]	Scoary	CVFS
Ciprofloxacin	116173	535	1090	20543	39
Ceftazidime	122985	563	919	17280	22
Gentamicin	113172	514	649	13992	7
Amoxicillin/Clavulanic acid	89981	411	1825	9306	67
Cefotaxime	109555	510	630	17604	21
Ampicillin	118695	540	866	13458	27
Cefuroxime	90946	406	1183	11675	34
Amoxicillin	59843	254	842	6491	15
Tobramycin	85460	394	557	9513	5
Trimethoprim	82725	360	658	9316	10

[1]The number of gene clusters after excluding all-zero gene clusters from the drug presence and absence table (i.e. gene clusters not present in any of the strains for this drug).

[2]Known AMR gene clusters predicted by CARD after excluding all-zero gene clusters from the drug presence and absence table.

[3]Results yielded by applying XGBoost feature selection algorithm on the drug datasets.

Supplementary Table S6. Distribution of the functional annotations of the CVFS-genes extracted from *Salmonella enterica* and *Escherichia coli*. The order of the gene functions is based on the sum of each functional annotation in descending order.

Available as Excel supplementary file.

Supplementary Table S7. Well-known AMR genes identified for different drug resistances.

	Drug	AMR genes[a]
S. enterica	Amoxicillin/Clavulanic acid	*bla*$_{CMY-2}$
	Ampicillin	*bla*$_{CMY-2}$, *bla*$_{CARB-3}$, *bla*$_{TEM-1}$
	Cefoxitin	*bla*$_{CMY-2}$
	Ceftiofur	*bla*$_{CMY-2}$
	Ceftriaxone	*bla*$_{CMY-2}$
	Chloramphenicol	*cmlA1*, *floR*
	Gentamicin	*ant(3'')-IIa, aph(6)-Id, ant(2'')-Ia,* *bla*$_{TEM-1}$
	Streptomycin	*aph(3'')-Ib, ant(3'')-IIa, aph(6)-Id*
	Sulfisoxazole	*sul1, sul2*
	Tetracycline	*tetA*, *tetB*
E. coli	Ceftazidime	*bla*$_{CMY-174}$, *bla*$_{CTX-M-15}$, *bla*$_{CTX-M-27}$, *bla*$_{SHV-66}$
	Gentamicin	*ant(3'')-IIa, aac(3)-IIe, ant(2'')-Ia*
	Amoxicillin/Clavulanic acid	*bla*$_{CMY-174}$, *bla*$_{OXA-1}$, *bla*$_{TEM-1}$
	Cefotaxime	*bla*$_{CMY-174}$, *bla*$_{CTX-M-15}$, *bla*$_{CTX-M-27}$, *bla*$_{SHV-66}$
	Ampicillin	*bla*$_{CMY-174}$, *bla*$_{CTX-M-15}$, *bla*$_{CTX-M-27}$, *bla*$_{OXA-1}$, *bla*$_{SHV-66}$, *bla*$_{TEM-1}$
	Cefuroxime	*bla*$_{CMY-174}$, *bla*$_{CTX-M-15}$, *bla*$_{TEM-1}$
	Amoxicillin	*bla*$_{OXA-1}$, *bla*$_{TEM-1}$
	Tobramycin	*aac(3)-IIe, aac(6')-Ib7*
	Trimethoprim	*dfrA1, dfrA8, dfrA14, dfrA17*

[a]AMR genes belonging to the same antibiotic class

Supplementary Table S8. Proportions of different gene classes identified by the CVFS approach for *Salmonella enterica*.

Drug	Known AMR gene %	Hypothetical proteins %	Mobile element %
Amoxicillin/ Clavulanic acid	100%	0%	0%
Ampicillin	19.05%	42.86%	19.05%
Cefoxitin	20%	60%	0%
Ceftiofur	100%	0%	0%
Ceftriaxone	25%	50%	25%
Chloramphenicol	28.57%	21.43%	28.57%
Gentamicin	57.14%	0%	28.57%
Streptomycin	9.43%	71.70%	3.38%
Sulfisoxazole	37.5%	25%	25%
Tetracycline	29.41%	47.06%	11.76%

Supplementary Table S9. Proportions of different gene classes identified by the CVFS approach for *Escherichia coli*.

Drug	Known AMR gene %	Hypothetical proteins %	Mobile element %
Amoxicillin/ Clavulanic acid	7.45%	52.24%	20.90%
Amoxicillin	20%	46.67%	13.33%
Ampicillin	25.93%	22.22%	22.22%
Cefotaxime	23.81%	23.81%	28.57%
Ceftazidime	27.27%	13.64%	27.27%
Cefuroxime	8.82%	64.71%	17.65%
Ciprofloxacin	5.13%	28.21%	17.95%
Gentamicin	57.14%	42.86%	0%
Tobramycin	60%	40%	0%
Trimethoprim	60%	0%	10%

Supplementary Table S10. Numbers of selected gene clusters and SVM prediction accuracies (in terms of AUROC) by different approaches for the *Salmonella enterica* resistances against five drugs, including spectinomycin, trimethoprim, sulphonamides, nalidixic acid, and kanamycin.

		All genes	XGB	CVFS
Gene num	Spectinomycin	9656	140	8
	Trimethoprim	11971	142	9
	Sulphonamides	10104	126	4
	Nalidixic acid	48981	558	49
	Kanamycin	24578	192	10
AUROC	Spectinomycin	81%	85%	93%
	Trimethoprim	79%	90%	94%
	Sulphonamides	79%	94%	66%
	Nalidixic acid	82%	92%	81%
	Kanamycin	82%	89%	89%

Supplementary Table S11. Proportions of hypothetical genes and mobile element-related genes for *S. enterica* genes selected by the XGBoost method without cross-validated feature selection approach.

	Hypothetical	Mobile element-related proteins
ampicillin	59.26%	13.79%
streptomycin	63.06%	11.82%
tetracycline	55.43%	12.90%
chloramphenicol	54.31%	13.79%
gentamicin	57.81%	13.75%
sulfisoxazole	57.26%	10.79%
ceftriaxone	61.98%	13.54%
ceftiofur	64.32%	13.57%
amoxicillin/clavulanic acid	51.97%	14.17%
cefoxitin	63.27%	10.62%

Supplementary Table S12. Proportions of known *S. enterica* AMR genes for genes selected by the XGBoost method without cross-validated feature selection approach.

	Known AMR genes (%)
ampicillin	2.06%
streptomycin	1.62%
tetracycline	2.49%
chloramphenicol	2.30%
gentamicin	4.38%
sulfisoxazole	3.73%
ceftriaxone	2.08%
ceftiofur	2.01%
amoxicillin/clavulanic acid	5.51%
cefoxitin	0.88%
